# MicroRNA Networks in Mouse Lung Organogenesis

**DOI:** 10.1371/journal.pone.0010854

**Published:** 2010-05-26

**Authors:** Jie Dong, Guoqian Jiang, Yan W. Asmann, Sandra Tomaszek, Jin Jen, Thomas Kislinger, Dennis A. Wigle

**Affiliations:** 1 Division of General Thoracic Surgery, Mayo Clinic Cancer Center, Mayo Clinic, Rochester, Minnesota, United States of America; 2 Division of Biomedical Statistics and Informatics, Department of Health Sciences Research, Mayo Clinic, Rochester, Minnesota, United States of America; 3 Pulmonary Medicine, Mayo Clinic Cancer Center, Mayo Clinic, Rochester, Minnesota, United States of America; 4 Department of Medical Biophysics, University of Toronto, Toronto, Ontario, Canada; 5 Ontario Cancer Institute, Princess Margaret Hospital, Toronto, Ontario, Canada; Cincinnati Children's Hospital Medical Center, United States of America

## Abstract

**Background:**

MicroRNAs (miRNAs) are known to be important regulators of both organ development and tumorigenesis. MiRNA networks and their regulation of messenger RNA (mRNA) translation and protein expression in specific biological processes are poorly understood.

**Methods:**

We explored the dynamic regulation of miRNAs in mouse lung organogenesis. Comprehensive miRNA and mRNA profiling was performed encompassing all recognized stages of lung development beginning at embryonic day 12 and continuing to adulthood. We analyzed the expression patterns of dynamically regulated miRNAs and mRNAs using a number of statistical and computational approaches, and in an integrated manner with protein levels from an existing mass-spectrometry derived protein database for lung development.

**Results:**

In total, 117 statistically significant miRNAs were dynamically regulated during mouse lung organogenesis and clustered into distinct temporal expression patterns. 11,220 mRNA probes were also shown to be dynamically regulated and clustered into distinct temporal expression patterns, with 3 major patterns accounting for 75% of all probes. 3,067 direct miRNA-mRNA correlation pairs were identified involving 37 miRNAs. Two defined correlation patterns were observed upon integration with protein data: 1) increased levels of specific miRNAs directly correlating with downregulation of predicted mRNA targets; and 2) increased levels of specific miRNAs directly correlating with downregulation of translated target proteins without detectable changes in mRNA levels. Of 1345 proteins analyzed, 55% appeared to be regulated in this manner with a direct correlation between miRNA and protein level, but without detectable change in mRNA levels.

**Conclusion:**

Systematic analysis of microRNA, mRNA, and protein levels over the time course of lung organogenesis demonstrates dynamic regulation and reveals 2 distinct patterns of miRNA-mRNA interaction. The translation of target proteins affected by miRNAs independent of changes in mRNA level appears to be a prominent mechanism of developmental regulation in lung organogenesis.

## Introduction

MiRNAs are a class of small RNAs (∼21–24 nt) that regulate the expression of target genes at the post-transcriptional level. They are first transcribed from miRNA genes in the genome as primary miRNA (pri-miRNAs) and then processed by an RNase III enzyme, Drosha, into premature miRNAs (pre-miRNAs) with hairpin structures. With the help of Exportin 5, pre-miRNAs are then transported into the cytoplasm, where they are cleaved by another RNase III enzyme, Dicer. The cleavage results in double-stranded duplexes. Usually, one strand of a duplex becomes the mature miRNA [1]. Mature miRNAs are then recruited into nucleoprotein complexes called RNA-induced silencing complexes (RISC). Based on the pairing of miRNAs and their target sites, the complexes can inhibit translation by either degradation of the messenger RNAs (mRNAs), or by blocking translation without degrading the targets [1], [2].

Individual miRNAs may target multiple mRNAs. Conversely, individual mRNAs may contain sequences complementary to multiple miRNA family members [3], [4]. It is estimated that miRNAs may be responsible for regulating the expression of nearly one-third of the human genome [5]. MiRNAs are known to play multiple roles in carcinogenesis, immune responses and organ development [6], [7], and have been implicated in many critical cellular processes, including apoptosis, proliferation, and differentiation [8]. Despite the identification of more than 800 mature human miRNAs and 700 mouse miRNAs, much remains to be discovered about their functional targets and biologic role.

The development of the mouse lung is initiated at embryonic day 9.5(E9.5), followed by the morphologically characterized pseudoglandular (E11.5–16.5), canalicular (E16.5–17.5), saccular (E17.5–P5) and alveolar stages (P5–30). The primitive airways begin as a ventral outpouching of foregut epithelium, with almost immediate branching to form the two main stem bronchi. Interactions between the surrounding mesenchyme and the developing airway epithelium function to promote further branching morphogenesis through the pseudoglandular and canalicular stages up to E17.5. Alveolarization begins in the saccules of the lung in parallel with development of the alveolar capillary bed, and proceeds up to completion at approximately 1 month of age [9], [10]. As in the organogenesis of many structures, formation of the lung is dependent on a myriad of interactions among signaling molecules and their receptors that mediate cell proliferation, differentiation, and other diversified functions under the control of complex regulatory networks [9].

Genetically engineered mouse mutant models using gene knockout [11], conditional alleles [12], [13], or transgenic misexpression [14] have all been used to gain insight into the specific genetic pathways controlling lung morphogenesis. Conditional ablation of Dicer in the mouse lung produces an abnormal phenotype with a dramatic reduction in branching morphogenesis [15]. Mice with deletion of the miR17-92 cluster die shortly after birth with lung hypoplasia and cardiac anomalies [16]. It is unknown if other individual or microRNA clusters exist with significant roles in lung organogenesis.

A number of miRNA profiling studies evaluating mouse and rat lung development have recently emerged and demonstrated the dynamic regulation of miRNAs during lung organogenesis. Williams et al performed miRNA profiling at 3 time points (P1, P14 and P60) of the developing mouse lung using a panel of 156 individual miRNAs [17]. They demonstrated that the overall expression profile was similar for mouse and human tissue, suggesting evolutionary conservation of miRNA expression during lung development. Lu et al performed miRNA cloning and sequencing at 2 time points (E11.5 and E17.5) and identified differences in the relative abundance of miRNAs across the 2 time points [18]. Further transgenic experiments suggest that miRNAs encoded by the miR-17-92 cluster positively promote proliferation of epithelial progenitor cells and inhibit their differentiation [19]. Bhaskaran et al performed miRNA profiling at 7 time points in rat lung development using an in-house platform and identified 21 miRNAs in 4 clusters that showed significant changes in expression [20]. No studies yet reported have explored interactions in the regulatory control of mRNA modules by individual or groups of miRNAs.

The purpose of this study was to identify and systematically explore dynamically regulated miRNAs and potential miRNA-mRNA interaction networks specific to mouse lung development. We performed comprehensive miRNA and mRNA profiling over a 7-point time course, encompassing all recognized stages of lung development beginning at embryonic day 12 and continuing to adulthood. We also systematically predicted potential direct mRNA targets of miRNAs through both miRNA-mRNA and miRNA-protein correlations. The results demonstrate evidence for two distinct patterns of miRNA-mRNA interaction, and reveal that translation of target proteins affected by miRNAs, independent of changes in mRNA level, appears to be a prominent mechanism of developmental regulation in lung organogenesis.

## Methods

### Preparation of timed-pregnant mice and isolation of total RNA

Approval of the study protocol was obtained from The Mayo Clinic Institutional Animal Care and Use Committee. All mice were maintained in a specific-pathogen-free animal facility at Mayo Clinic and all animal experiments were carried out according to the provisions of the Animal Welfare Act, PHS Animal Welfare Policy, and the principles of the NIH Guide for the Care and Use of Laboratory Animals. ICR female mice were bred for timed pregnancies. The day of plug observation was considered day 0 of gestation. Lungs were isolated by manual dissection with the aid of a dissecting microscope where necessary. Lungs were washed with PBS. Total RNA was isolated from pooled whole lungs using Trizol (Invitrogen) for mRNA expression microarray and mir VanaTM miRNA Isolation Kit (Ambion) for ABI microRNA real-time PCR array according to the manufacturer's instructions. RNA quality and integrity was confirmed by denaturing gel electrophoresis.

### mRNA and miRNA arrays

The mRNA expression profiling was performed using the Affymetrix GeneChip Mouse Genome 430 2.0 Array containing probes to query more than 39,000 transcripts. The reverse transcription, labeling and hybridization of mRNA were performed in the Mayo Microarray Shared Resource. The data has been submitted to the Gene Expression Omnibus (GEO) database with accession number GSE20954.

MiRNA expression profiling was performed using the Taqman Rodent MicroRNA Array Card A and Card B (Applied Biosystems) containing all 521 mature mouse miRNAs in miRBase 10.1 http://microrna.sanger.ac.uk. In brief, miRNA was reverse transcribed to cDNA using the Megaplex TM RT Rodent Primers Pool and the TaqMan MicroRNA Reverse Transcription Kit. Quantitative 384 well TaqMan Low Density Array real-time PCR was run on the ABI PRISM 7900 System using the TaqMan Universal PCR Master Mix. Raw miRNA array data was analyzed by using RQ manager software on the ABI system. All undetectable data and the data with C_T_ values greater than 35 were treated as 35 [21]. Normalized C_T_ (ΔC_T_) was calculated by comparing each miRNA value to that of small nuclear U6 RNA. The U6 RNA is a common internal control for each microRNA array card. The copy number of miRNAs in each cell (assuming each cell contains 30 pg of total RNA) was calculated from a formula 10^(40-C^_T_^)/3.34^/22 that was estimated using synthetic lin-4 miRNA [22]. The data has been submitted to the GEO database with accession number GSE21052.

### Data processing and analysis

Both mRNA and miRNA array data analysis was performed with the Partek Genomics Suite 6.4 software. For mRNA expression data, Affymetrix CEL files were imported. The data were normalized with the Robust Multichip Average Algorithm [23] and converted to log_2_ values. For miRNA expression data, the ΔC_T_ value was directly imported as a log_2_ value. The greater the ΔC_T_ value, the lower the miRNA expression value (i.e. copy number). Log data were used for hierarchical clustering and statistical analysis.

The normalized data were subsequently analyzed by principal component analysis (PCA) to determine if any intrinsic clustering or outliers existed within the data set. A combination of descriptive statistics such as Min/Max (i.e. minimum/maximum; we use minimum for miRNA as the greater the copy number the smaller the expression value), Range (the difference between maximal and minimal values across 7 time points) and the False Discovery Rate (FDR) adjusted p-values derived from statistical analysis were used to identify significantly altered miRNAs and mRNA probes.

We analyzed the time course data using 2 different statistical approaches, ANOVA and a time-course specific statistical method implemented in an open source software program, the Extraction of Differential Gene Expression (EDGE, available at http://www.genomine.org/edge/) [24]. The cut-off values of the descriptive statistics were empirically set up at a strict level to minimize off-target correlations derived from ANOVA. A comparative analysis using the EDGE approach was performed to exclude observations made on the basis of a single statistical approach.

Hierarchical cluster analyses were performed using dChip software (www.dchip.org) on filtered datasets to identify temporal expression patterns of miRNA and mRNA levels in mouse lung development.

Pearson correlation analysis was performed between two sets of time-course expression values for significant miRNAs and mRNA probes to identify negatively correlated miRNA-mRNA pairs within certain cut off values. The computational mRNA targets of each significant miRNA retrieved from the miRBase (version 5) and TargetScanMouse (5.1) databases were used for validation, with the assumption that overlaps between negatively correlated mRNA targets and computationally detected mRNA targets may imply direct mRNA targets for each miRNA. g:Profiler was used to convert Transcript IDs from miRBase into Affymetrix mouse430 Probe Set IDs [25].

A protein profiling dataset generated by shotgun mass spectrometry from a previous study [26] was used to explore microRNA-protein correlations. The protein profiling data is a time course study of protein expression levels with 6 time points, encompassing the protein data from early embryonic stage E13.5 through adulthood during ICR mouse lung development using gel-free two-dimensional liquid chromatography coupled to shotgun tandem mass spectrometry (MudPIT). We integrated the expression values of each protein detected from three organelle fractions (i.e. nuclear, mitochondrion, cytosol fractions) into a unified value and then converted these into log_2_ values. Pearson correlation analysis was performed between the time-course expression values of significant miRNAs and the protein data across 6 time points with a mapping of the two time courses (E14 to E13.5, E16 to E16, E18 to E18, P2 to P2, P10 to P14 and Adult to Adult). g:Profiler was used to convert the protein UNIProtKB IDs into the Affymetrix mouse 430 Probe Set IDs. A similar approach was used to identify the direct miRNA targets through comparison with the miRBase file.

### Gene ontology biological process and pathway analysis

Gene ontology (GO) biological process annotations and pathway annotations (GenMapp, KEGG) were performed against Affymetrix annotation files using Partek software. We performed GO analysis and pathway analysis to gain insight into biological processes and pathways in lung development among different expression patterns of mRNAs, among direct mRNA targets of different expression patterns of miRNAs, and among direct mRNA targets of each miRNA.

### Genome localization of miRNAs

The UCSC Genome Brower (genome.ucsc.edu) was used to visualize and analyze the chromosome localization for total mouse miRNAs and significant miRNAs involved in lung development.

## Results

**Figure 1:**
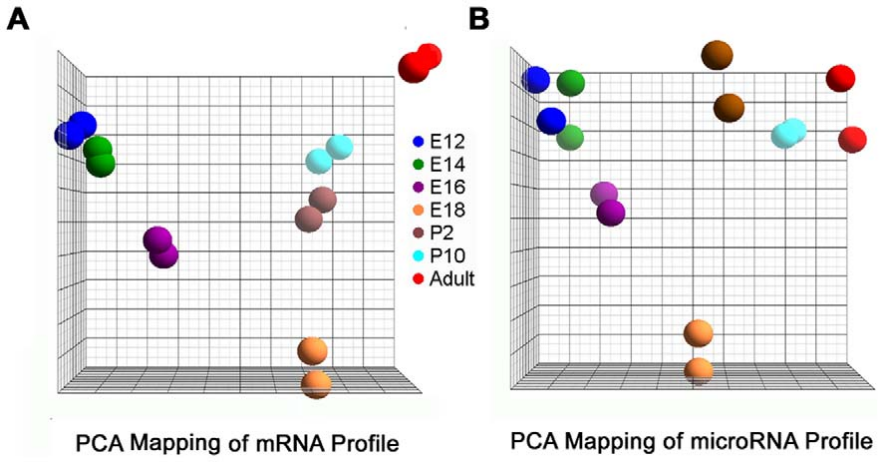
Scatter plots of miRNA and mRNA data by Principle Component Analysis (PCA). (A) Expression array data for mRNA. (B) Expression array data for miRNA. Samples are colored by different mouse lung development time points. The same color represents the replicate samples.

In total, the expression values of 45,101 probes for mRNA profiling in 7 time points (2 samples in each time point) were obtained after normalization. The normalized expression values of 521 miRNAs in the same 7 time points (2 samples in each time point) were also obtained. The 3-D graphical visualization of the principal component (PCA) analysis for both mRNA and miRNA expression values from replicate samples in each time point have similar patterns (Figure 1).

Three criteria were used for identifying mRNA probes and miRNAs which changed significantly in expression level during different time points of lung development. For mRNA expression data, the cut-off value for the descriptive statistic Max was set as >7, and the cut-off value of Range as >1.2 (i.e. fold change>2.3). The p value for ANOVA analysis was set as p<0.05 and further adjusted by FDR (Step Up, FDR<0.05) to p<0.03. From these filters, 11,220 probes were identified as dynamically regulated across the time course. Using the EDGE statistical approach, 11,118 probes were identified as dynamically regulated across the time course. The overlap between the 2 approaches was 11,046 probes in total, or 98.45% of all probes identified by ANOVA.

For miRNA expression data, a cut-off value for Min was set as ΔC_T_ <10 and a cut-off value for Range was >2.2 (i.e. fold change>4.6). The p value for ANOVA was set as p<0.05 and further adjusted by FDR (FDR<0.05) to p<0.017. In total, 117 dynamically regulated miRNAs were identified through these filters. Using the EDGE approach, 121 dynamically regulated miRNAs were identified, with 116 (99.15%) overlapping with those identified by ANOVA. Of these 116 miRNAs, miR-466d-3p (1520 fold), miR-449a (975 fold), miR-29a (479 fold), miR-146b (278 fold) and miR-409-3p (255 fold) were the top five miRNAs which had the highest fold changes across the dataset. miR-126-3p, miR-24, miR-16, miR-19b, and miR-17 were the top five miRNAs with the highest absolute expression values. Of these, MiR-126-3p has the highest expression value with ΔC_T_ value 0.14 (i.e. copy number 159299).

### mRNA expression

For characterizing the expression patterns of lung development, hierarchical clustering analysis was performed to generate a dendrogram of both mRNA and miRNA data sets that met the above criteria.

**Figure 2:**
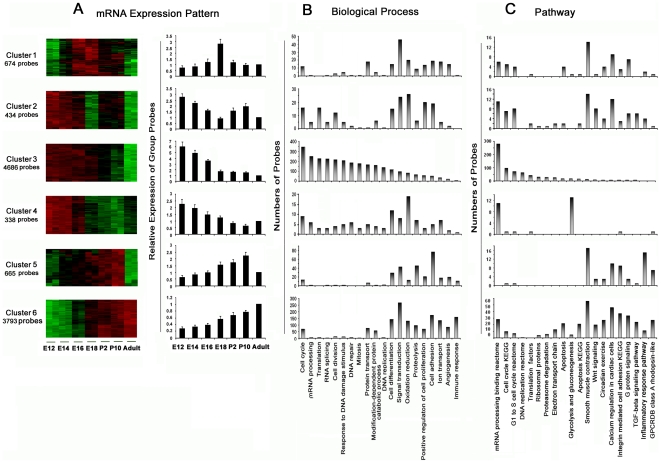
Temporal expression patterns of mRNA using hierarchical cluster analysis and biological functional patterns by GO biological process and pathway analysis. (A) 6 temporal expression patterns represented by color heat maps and bar graphs (x axis indicating the 7 time points, y axis indicating the relative expression value of group probes). The relative expression values in each time point are the ratios normalized against those in the adult group. (B) Biological process patterns of 6 expression clusters represented by bar graphs (x axis indicating top 20 GO terms on biological process, y axis indicating the number of probes) (C) Pathway patterns of 6 expression clusters represented by bar graphs (x axis indicating top 20 GenMAPP pathway annotations, y axis indicating the number of probes).

Through analysis of the dendrogram for 11,220 mRNA probes, we identified 6 temporal expression patterns (i.e. Cluster 1–6 in Figure 2A) across 7 time points accounting for 94% of all significant probes. Clusters 3 and 6 cover the majority of probes, accounting for approximately 76% of the total. Cluster 3 shows that expression values of 4686 (42%) mRNA probes are high at the early embryonic stage (E12), decrease gradually through E16, and then decrease dramatically at the late embryonic stage (E18) and through postnatal and adult stages. Cluster 6 shows that expression values of 3793 (34%) mRNA probes are low at the early embryonic stage (E12) and increase gradually through all stages and reach a peak at the adult stage. Cluster 1 demonstrates those expression values of 674 (6%) probes peaking at the late embryonic stage (E18) and decreasing dramatically after birth through adult stages. The remaining 3 clusters, accounting for 12% of total probes, show patterns distinct from the above 3 patterns.

Functional analysis using Gene Ontology annotation was performed. In all clusters, the genes are enriched in categories of “transcription”, “regulation of transcription”, “transport”, “multicellular organismal development”, “protein amino acid phosphorylation” and “metabolic process”. Excluding these common functions, the top 20 GO terms ranked by the number of probes display distinct enrichment profiles across different clusters (Figure 2B). For Cluster 3 in which genes are active in the early embryonic stage of lung development, enrichment occurs for genes involved in “cell cycle”, “mRNA processing”, “translation”, “RNA splicing”, “cell division”, “response to DNA damage stimulus”, “DNA repair”, “mitosis” and “DNA replication”. This is in contrast to Cluster 6 where genes are predominantly active in adult stages, with enrichment in genes involved in “signal transduction”, “cell differentiation”, “immune response”, “cell adhesion”, “oxidation reduction”, and “angiogenesis”.

Pathway analysis using GenMAPP and KEGG annotations was performed for all 6 clusters. Figure 2C shows a comparison of the top 20 pathways ranked by the number of genes. No common pathway was identified across all 6 clusters. For Cluster 3, genes are enriched for pathways “mRNA processing binding reactome”, “cell cycle KEGG”, “G1 to S cell cycle reactome”, and “DNA replication reactome”; whereas for Cluster 6, genes are enriched in pathways “smooth muscle contraction”, “G protein signaling”, “integrin-mediated cell adhesion KEGG”, “TGF beta signaling pathway” and “Wnt signaling”. For Cluster 1, genes are enriched rich for “smooth muscle contraction” and “G protein signaling”.

### miRNA expression

**Figure 3:**
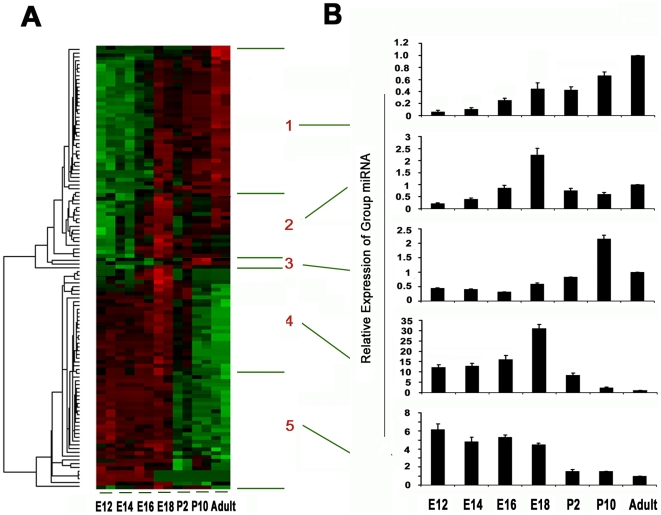
Temporal expression patterns of miRNA using hierarchical cluster analysis. (A) 5 temporal expression patterns represented by color heat maps. (B) 5 temporal expression patterns represented by bar graphs (x axis indicating the 7 time points, y axis indicating the relative expression values of group miRNAs). With miRNA copy number as an miRNA expression value, The relative expression values in each time point are the ratios normalized against those in the Adult group.

Through analysis of the cluster dendrogram for 117 dynamically regulated miRNAs, we identified 5 temporal expression patterns from two major pattern groups across 7 time points. For the first pattern group, the expression values are low at early embryonic stages and increasing to reach a peak at late embryonic or postnatal and adult stages. There are 61 miRNAs in this group, in which 3 distinct temporal expression patterns (i.e. Clusters 1–3 in Figure 3) were identified. Cluster 1 show that the expression values of miRNAs increase gradually through all stages and reach a peak at the adult stage (P30), whereas Cluster 2 peaks at the late embryonic stage (E18) and Cluster 3 at the postnatal stage (P10).

For the second pattern group, the expression values are high at embryonic stages and decrease dramatically at the postnatal and adult stage. There are 56 total miRNAs in this group, in which 2 temporal expression patterns (i.e. Clusters 4–5 in Figure 3) were identified. Cluster 5 shows the expression values of miRNAs decrease gradually through the embryonic stages and then decrease dramatically after birth through the adult stage, whereas Cluster 4 peaks at the late embryonic stage (E18) and then decreases dramatically.

### Directly correlated target mRNAs for miRNA clusters

#### 1) Identified by mRNA expression

We performed pair-wise Pearson correlation analysis across 7 time points to evaluate potential correlations between 117 miRNA and 11,220 mRNA expression levels. With setting a cut off value as coefficient >|0.9|, we detected 69,725 miRNA-mRNA pairs with negative correlation and 47,532 pairs with positive correlation. We found that for each miRNA, the greater the number of negatively correlated pairs, a corresponding greater number of positive correlation pairs also existed. Interestingly, for those miRNAs in Cluster 2, 3 and 4, almost no correlated pairs were identified. In Cluster 5, about 2/3 of miRNAs do not have correlated pairs identified. Table S1 shows the correlation analysis results of all 117 miRNAs which are sorted by clusters and number of probes.

To test if the miRNA-correlated mRNA genes are direct miRNA targets, we downloaded the miRBase database and compared the computational targets of each miRNA with the miRNA negatively correlated mRNA probes. In total, 148,840 miRNA-mRNA probe pairs were identified from the miRBase database. 3,067 unique pairs were filtered out as direct miRNA targets with coefficient <−0.9 (Table S1). We found that all of the miRNAs having direct targets identified are distributed in Cluster 1 and 5. In total, 80 out of 117 miRNAs did not have direct targets identified as they did not have negatively correlated mRNAs. For those miRNAs in Cluster 2, 3 and 4, no direct targets were identified. About 2/3 of miRNAs in Cluster 5 do not have direct targets identified.

For comparison with miRBase, the TargetScan database was also used to predict direct miRNA targets. In total, 330 direct miRNA targets were filtered out and only 7 miRNAs had direct targets identified. All of them are in cluster 1 and 5 (Table S1), indicating that the TargetScan database is more stringent with potentially lower sensitivity than miRBase.

#### 2) Identified by protein expression

We performed pair-wise Pearson correlation analysis across 6 time points to evaluate potential correlations between the expression levels of 117 miRNAs and 3330 UniProtKB proteins. After mapping the UNIProtKB IDs of 3330 proteins onto 6326 Affymetrix mouse430 Probe Set IDs and setting the cut off value as coefficient >|0.8|, we detected 41,774 miRNA-protein pairs with negative correlation. By comparing the miRBase computational targets of each miRNA/mRNA pair with the miRNA/negatively correlated protein pair, 1345 unique miRNA/protein pairs were identified as direct miRNA targets (Table S2). Most of the identified miRNAs had direct mRNA targets identified except for three, indicating that correlation with protein levels has a higher yield of computationally predicted mRNA targets versus those obtained by mRNA profiling alone (97.4% versus 31.6%). Interestingly, 54.9% of the mRNA probes identified as direct targets through protein expression data do not belong to the significantly changed subset (n = 11220) of mRNAs.

### Genome localization of miRNAs

**Figure 4:**
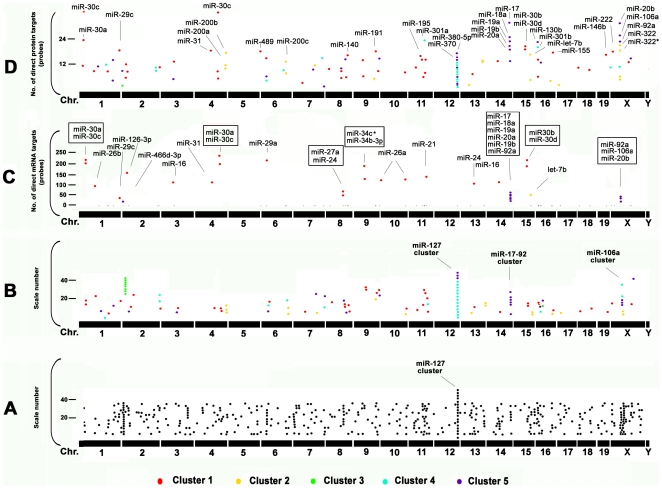
Visualization of chromosome localization of mouse miRNAs. Chromosome localization of (A) total 521 mouse miRNAs (Sanger version 10.1) and (B) 117 significant miRNAs in different clusters (marked in different colors) involved in lung development. The scale is given to make those miRNAs having the same or very close chromosome location displayed in a vertical line. (C) Numbers of direct mRNA targets for each miRNA detected by miRNA/mRNA correlation. 30 miRNAs with multiple direct targets are annotated. MiRNAs within clusters are boxed. (D) Numbers of direct protein targets detected for each miRNA by miRNA/protein correlation. The top 30 miRNAs are annotated.

MiRNAs are found throughout mammalian genomes. Half of the known miRNAs are located inside or close to fragile sites which are genomically unstable and common breakpoints associated with cancer. Nearly 40% of miRNAs exist in genomic clusters. Some clusters reflect the processing of a number of miRNAs from a single large polycistronic transcript such that presumably all of the miRNAs are under control of the same promoter and in the same transcriptional orientation [27]. With the stringent criteria that a cluster should consist of more than two members positioned within 1 Mb, there are 22 miRNA clusters identified in mouse [27], [28], [29]. The largest is the miR-127 cluster with greater than 50 members on mouse chromosome 12 (Chr.12). Other large clusters include the miR-29a cluster on Chr.6, the miR-23a cluster on chr.8, the miR-17-92 cluster on Chr.14 and the miR-106a cluster on Chr.X (Figure 4A). Significant miRNAs dynamically regulated in lung development were positioned throughout the genome except for Chr.5 and Chr.Y (Figure 4B). 23 miRNAs belonging to the miR-127 cluster were increased, whereas all 6 miRNAs in the miRNA-17-92 cluster (mir-17, 18a, 19a, 19b-1, 20a, and 92-1) and 3 miRNAs (mir-20b, 90a-2 and 106a) in the miR-106a cluster that all belong to miRNA cluster 5 were dramatically decreased (Figure 4). Interestingly, almost all 23 miRNAs in the miR-127 cluster that have the same strand orientation belong to miRNA Cluster 4 and have highest expression around E18. Within the miR-127 cluster, no miRNA targets were identified by miRNA/mRNA pairing (Figure 4C), while all had direct mRNA targets when miRNA/protein pairs were analyzed, such as miR-380-5p, miR-370, and miR-434. This suggests that the miR-127 cluster may be involved in part of the regulation of the start of alveolar formation by inhibiting mRNA translation of specific targets without changes in mRNA levels. A number of further targets were identified through miRNA/protein correlations, such as the mir-200 family (miR-200a, miR-200b and miR-200c), miR-191, miR-195, miR-301, and miR-322 (Figure 4D).

### Pathway involvement for individual miRNAs

In this study, we found that a number of miRNA families are dynamically regulated during mouse lung development. This includes miRNA families miR-30 (miR-30a, miR-30d, miR-30e, miR-30b, miR-30c, miR-30e*), miR-24 (miR-24, miR-24-2*), miR-26 (miR-26a, miR-26b), miR-29 (miR-29a, miR-29c), miR-34 (miR-34b-3p, miR-34c*) in Cluster 1 which has high expression in the adulthood stage, and miR-20 (miR-20a, miR-20b) in cluster 5 which has high expression in the early stages of lung organogenesis. To computationally explore the potential functional relevance of dynamically regulated miRNAs during lung development, we employed biological process and pathway analysis. We found that miRNAs within the same family or same genome localization cluster have similar biological functions and appeared to be involved in similar pathways. For example, there are 5 miRNA members in the miR-30 family that are involved in TGF Beta signaling pathway through the gene “Tgfbr1” and 5 miRNAs (miR-17a, 18a, 20a, 20b, 92a) in miR-17-92 cluster that are involved the same pathway through the gene Smad6. There are 4 miRNAs (miR-30b, let-7b, 18a, and 19a) that are involved in the Wnt signaling pathway through the genes Fzd2, Racgap1, Myc and Prkce. A list of 30 miRNAs with their respective corresponding top 10 GO biological process terms, pathways and pathway-related target gene names is shown in Table S3.

## Discussion

In this study, we explored the dynamic regulation of microRNA, mRNA, and protein levels over the time course of lung organogenesis using a systematic genome-wide approach. First, we performed genome-wide mRNA and miRNA profiling across all recognized stages of lung development beginning at early embryonic stages and continuing to adulthood. Second, we identified the subset of mRNAs and miRNAs for which expression values changed significantly across the time course using statistical analysis. Thirdly, we clustered the significant subsets to identify their temporal expression patterns with GO functional enrichment and pathway analysis. Fourthly, we performed both miRNA-mRNA and miRNA-protein correlation analyses and integrated these with computational target predictions to study potential direct targets of miRNAs. Lastly, we explored the biological function and pathway roles of miRNAs involved in lung development through their mRNA or protein targets.

As part of the analysis, we combined temporal correlation analysis and computational target mapping to study the potential direct targets of miRNAs. As many miRNAs cause degradation of their mRNA targets and a large number of mRNAs are regulated in this manner, expression profiling patterns of miRNAs and their target mRNAs should reveal an inverse relationship or correlation [5], [30]. We considered that a negative correlation between miRNA and mRNA (or protein) expression levels would mimic such an inverse relationship. In this study, we chose the cutoff value of correlation coefficient as −0.9 for miRNA-mRNA correlations and −0.8 for miRNA-protein correlations, both of which represent a strong negative correlation. Combined with computational prediction, simultaneous profiling of miRNA and mRNA expression levels may be a strategy for the validation and identification of functional miRNA targets [31], [32]. Time-course studies provide further information that could readily be missed in a cross-sectional study based on a single time point [33].

The first point of our analysis was to understand if gene expression varied by time course, and in particular which genes were changing. To answer this question, we adopted an empirical model combining stringent descriptive statistics with FDR adjusted p-values derived from ANOVA analysis. We furthered this with a second analysis using the time-course specific EDGE methodology, demonstrating minimal difference between the 2 approaches. MicroRNA expression levels were analyzed in a similar manner. From this, we conclude that the correlations observed between mRNA, miRNA, and protein levels are valid from the standpoint of 2 different statistical approaches to the data.

Computational miRNA target prediction algorithms have been developed based on common features of known miRNAs and their mRNA target interactions and these have greatly facilitated the search for miRNA targets [34]. However, computational miRNA target prediction does not account for physiological factors that could influence the existence or outcome of the predicted miRNA-mRNA interaction, including the stoichiometry of miRNAs and targets, or the involvement of other regulatory proteins [35], [36]. Current computational prediction tools are diverse, both in approach and performance. It is difficult to decide which predicted miRNA-target interactions are more likely to be accurate and which tool provides the best performance [32], [34]. When considering current predictions that are available for mouse, we evaluated those from miRBase Targets [37], EIMMo[38], PicTar [39]and TargetScan [40], [41], all of which use site conservation as a prediction criterion. Comparisons with TargetScan and EIMMo require stringent seed pairing, while miRBase requires moderately stringent seed pairing. Although evaluation of proteomic changes after miRNA addition or deletion provides evidence that tools stringently requiring seed pairing perform better than those tools with more moderately stringent cutoffs [42], [43], perfect base pair matching does not guarantee interaction between specific miRNAs and target genes [35]. Wobble base pairs are often tolerated in target sites [44], [45]. For the current study, both miRBase and TargetScan were used to predict direct miRNA targets by comparing negatively correlated mRNA expression with computational predictions. 3,067 unique miRNA-mRNA pairs (involving 37 unique miRNAs) were identified using miRBase whereas 330 pairs (7 unique miRNAs) were identified using the TargetScan database. We considered it appropriate to use the miRBase target prediction database given the relatively higher prediction sensitivity, although it is not clear how much specificity is lost with this approach.

Initially, we examined miRNA-mRNA correlations to predict the direct targets of 117 dynamically regulated miRNAs. Surprisingly, 68.4% (80) of miRNAs in this group did not have any direct targets identified based on a correlation coefficient cutoff value of −0.9. To examine this further, we relaxed the cutoff values and found that 51.3% (60) of miRNAs with a cutoff value of correlation coefficient −0.8, and 33.3% (39) of miRNAs for a cutoff of −0.7, do not have direct targets identified (Table S1). The results imply that a large number of miRNAs dynamically regulated during lung organogenesis may not directly cause degradation of mRNA targets. As miRNAs have the potential to modulate protein translation independent of degrading the mRNA for a specific target, we analyzed our data in the context of a previously published protein database for lung development [26]. Looking for negative correlations between miRNA level and protein expression based on spectral counts, we found that a higher number of miRNAs, 114 (97.4%) had a direct target protein identified (Table S2). Importantly, 54.9% of mRNAs for which a corresponding direct target protein was identified do not belong to the subset of significantly varying mRNAs during lung development, implying that a number of miRNAs may effect decreased protein expression even though they do not cause detectable changes in mRNA level. In other words, a large number of protein level changes may occur that would not be apparent from the study of mRNA data alone.

The underlying mechanisms explaining these observations remain to be investigated. Selbach et al pointed out that whether the mRNA is cleaved or whether productive translation is inhibited depends on the complementarity of the miRNA to the mRNA. If the complementarity is not enough for cleavage but still involves some degree of binding, then translation will be repressed [42]. However, it has been demonstrated that although some targets are repressed without detectable changes in mRNA levels, those translationally repressed by more than a third also displayed detectable mRNA decreases, and, for the more highly repressed targets, mRNA degradation usually comprised the major component of repression. The impact of miRNAs on the proteome indicated that for most interactions miRNAs act as rheostats to make fine-scale adjustment to protein output [43]. Our data suggest there are at least two major functional patterns of miRNA regulation of gene and protein levels in mouse lung development: 1) directly downregulating mRNA levels; 2) directly repressing translation of genes without detectable changes in mRNA levels.

Individual miRNAs may target hundreds of mRNAs and individual genes may be regulated by a number of miRNAs or multiple members of same miRNA family. Individual miRNAs have predicted propensity to target genes with related functions which can provide insight into the biological roles of individual miRNAs [34], [38]. The function of the target genes for Cluster 1 miRNAs are highly related with cell proliferation as they are enriched for GO biological processes “cell cycle”, “mitosis”, “DNA replication” “DNA repair”, and “RNA splicing”. Specifically, we identified a number of genes such as E2F1, P53, c-Myc, CDK2, and others that regulate progenitor cell fate and play important roles in lung development and lung cancer [9], [10]. In addition, a number of studies have reported that c-Myc expression is repressed by let-7, that p53 interacts with miR-34, and that growth arrest can be induced by miR-34 through modulation of the E2F pathway in human colon cancer cells [46], [47], [48]. In Cluster 5, we noticed that cluster miR-17-92 and cluster miR-106a had high expression in early embryonic development of the lung then steady declines through the remainder of development and into adulthood. A number of studies have demonstrated the miRNAs in these two clusters play critical roles in lung development and lung cancer [16], [19], [49], [50]. The potential mechanisms involved may include activation of targets such as RbI2, E2F1-3, and PTEN that are all known cell cycle regulators [19], [51], [52]. However, Carraro et al reports that function of the miR-17-92 cluster may be to maintain the structural homeostasis of developing lung epithelium through the targets Mapk14 and stat3, as systematic inhibition of miR-17 did not produce an arrest of proliferation [53]. In this study, the direct mRNA targets of miRNA-17-92 appeared to be a group of genes that have lowest expression in early embryonic development and highest expression in adulthood. The expression pattern indicates that the cluster most likely plays a role in the later stages of lung development after lung branching morphogenesis is complete. GO biological process analysis shows that the function of these genes focus on “protein phosphorylation”, “metabolic process”, “signal transduction”, “intercellular signaling cascade”, “cell adhesion” and “angiogenesis”. Pathway analyses demonstrate that the miRNA-17-92 cluster is involved in G-protein signaling, the Wnt pathway through Prkce as well as TGF-beta pathway signaling through Smad6 (which is already confirmed by [54]).

Our results provide important insights into the global dynamics of miRNA networks and their mediation of mRNA translation into protein products. A number of limitations to the current study should be noted. One is the fact that the discovery and characterization of new miRNAs is an ongoing process. A further limitation is the fact that analyses of RNA and protein in this study were derived from bulk whole lung, and do not provide representation of the myriad of cell types and their organization present in the developing or adult lung. An important extension to the current study will be to localize the dynamically regulated miRNAs identified to specific regional niches within the developing lung.

In this study, we analyzed the expression patterns of dynamically regulated miRNA, mRNA and proteins during lung development. Furthermore, we developed a novel approach to systematically predict potential direct targets of miRNAs from the data through both miRNA-mRNA and miRNA-protein correlation analysis and computational target mapping. Systematic analysis of microRNA, mRNA, and protein levels over the time course of lung organogenesis demonstrates dynamic regulation and reveals 2 distinct patterns of miRNA-mRNA interaction: 1) increased levels of specific miRNAs directly correlating with downregulation of predicted mRNA targets; and 2) increased levels of specific miRNAs directly correlating with downregulation of translated target proteins without detectable changes in mRNA levels. The data also suggests that the translation of target proteins affected by miRNAs independent of changes in mRNA level appears to be a prominent mechanism of developmental regulation in lung organogenesis.

## Supporting Information

Table S1 Correlation analysis and direct target prediction results of 117 significant miRNAs by miRNA/mRNA correlations in mouse lung development.
(0.18 MB PDF)

Table S2 Correlation analysis and direct target predication results of 117 significant miRNA by miRNA/protein correlations in mouse lung development.
(0.12 MB PDF)

Table S3 A list of 30 miRNAs having direct targets identified by miRNA/mRNA correlation, with their respective corresponding top 10 GO biological process terms, all pathways and pathway-related target gene names.
(0.04 MB PDF)
