# Supplementary material for: MicroRNA Networks in Mouse Lung Organogenesis
Source: PLoS One. 2010 May 26;5(5):e10854. doi: 10.1371/journal.pone.0010854 (PMC2877109; doi:10.1371/journal.pone.0010854)
Supplement: Table S1 — Correlation analysis and direct target prediction results of 117 significant miRNAs by miRNA/mRNA correlations in mouse lung development. (0.18 MB PDF) [file pone.0010854.s001.pdf]

Table 1

A-Neg: Number of Negatively Correlated Targets (Coefficient cutoff<-0.7, -0.8 or -0.9))
A-Pos: Number of Positively Correlated Targets (Coefficient cutoff>0.9)
B: Number of Computational Targets from miRBase or TargetScan
C: Number of direct miRNA targets through Overlapping of B and A-Neg

miRNA	miRNA	A Neg(0.7)	A Neg(0.8)	A Neg(0.9)	A Pos(0.9)	B	C Neg(0.7)	C Neg(0.8)	C Neg(0.9)	B	C Neg(0.7)	C Neg(0.8)	C Neg(0.9)
Cluster						miRBase	miRBase	miRBase	miRBase	TargetScan	Targetscan	Targetscan	Targetscan
1	mmu-miR-30e	5175	4814	4015	2603	1528	281	273	236	0	0	0	0
1	mmu-miR-30a	5140	4779	3832	2309	1467	275	263	226	0	0	0	0
1	mmu-miR-29a	5171	4811	3692	2568	1576	269	256	213	0	0	0	0
1	mmu-miR-30d	5178	4837	3943	2467	1432	254	243	206	0	0	0	0
1	mmu-miR-30c	5029	4596	3429	2049	1510	261	237	196	0	0	0	0
1	mmu-miR-30b	5109	4655	3253	2030	1517	271	258	187	0	0	0	0
1	mmu-miR-34b-3p	5153	4800	3839	2616	1254	209	200	182	0	0	0	0
1	mmu-miR-126-3p	5193	4846	4035	2701	1234	192	185	154	5	3	2	1
1	mmu-miR-21	5080	4690	3433	2231	1148	179	172	139	0	0	0	0
1	mmu-miR-34c*	4951	4363	2821	1666	983	181	172	129	0	0	0	0
1	mmu-miR-26a	5084	4639	3155	2067	1287	182	176	125	0	0	0	0
1	mmu-miR-16	4990	4456	2830	1930	1380	189	175	111	0	0	0	0
1	mmu-miR-30e*	5054	4561	3094	1831	1245	185	169	111	0	0	0	0
1	mmu-miR-31	5165	4774	3382	2097	1203	171	162	111	199	90	85	60
1	mmu-miR-24	5033	4484	2829	1908	1220	158	150	103	305	126	112	76
1	mmu-miR-26b	4876	4185	2208	1535	1254	186	169	95	570	255	214	97
1	mmu-miR-30a*	5026	4537	2523	1494	1253	172	157	74	0	0	0	0
1	mmu-miR-24-2*	5062	4578	2531	1631	1165	133	124	67	0	0	0	0
1	mmu-let-7b	4861	3696	913	728	1459	177	137	46	0	0	0	0
1	mmu-miR-27a	4996	4354	1887	1285	1267	160	141	45	0	0	0	0
1	mmu-miR-29c	4218	2441	696	762	1557	250	140	36	609	210	128	54
1	mmu-miR-150	3992	723	11	1	1240	110	33	1	135	47	10	0
1	mmu-miR-195	2665	194	6	1	1229	121	9	1	0	0	0	0
1	mmu-miR-486	2393	135	5	1	1436	95	5	1	106	16	0	0
1	mmu-miR-126-5p	3273	301	8	1	1103	111	4	0	0	0	0	0
1	mmu-miR-133a	242	15	4	0	1356	11	0	0	0	0	0	0
1	mmu-miR-140	79	9	2	0	1281	1	0	0	0	0	0	0
1	mmu-miR-142-3p	861	48	5	1	1310	28	1	0	197	8	0	0
1	mmu-miR-145	1906	85	6	1	1266	81	3	0	428	83	3	0
1	mmu-miR-146a	3517	413	10	1	1423	150	23	0	0	0	0	0
1	mmu-miR-146b	3325	196	9	2	1375	158	7	0	115	25	3	0
1	mmu-miR-155	152	10	4	1	1227	2	0	0	223	0	0	0
1	mmu-miR-181a	143	13	2	0	1374	3	0	0	0	0	0	0
1	mmu-miR-191	811	47	5	1	1238	36	1	0	24	0	0	0

1	mmu-miR-222	3751	423	12	3	1182	135	19	0	254	81	8	0
1	mmu-miR-223	2420	149	6	1	1220	107	8	0	186	42	0	0
1	mmu-miR-328	185	12	4	0	1115	5	0	0	77	1	0	0
1	mmu-miR-365	680	37	5	1	1385	36	0	0	160	4	0	0
1	mmu-miR-451	1136	74	4	1	1278	56	5	0	3	0	0	0
1	mmu-miR-484	195	12	5	0	1199	5	0	0	0	0	0	0
1	mmu-miR-489	209	12	5	1	1241	11	0	0	102	5	0	0
1	mmu-miR-667	25	6	2	0	1283	1	0	0	0	0	0	0
2	mmu-let-7e	1209	233	9	44	1310	42	10	1	0	0	0	0
2	mmu-let-7g	1712	338	21	35	1406	70	17	1	0	0	0	0
2	mmu-miR-200b	885	66	4	0	1601	43	4	1	0	0	0	0
2	mmu-let-7c	2136	368	11	14	1459	90	17	0	0	0	0	0
2	mmu-let-7d	558	118	5	21	1377	26	5	0	0	0	0	0
2	mmu-let-7i	615	133	5	32	1451	22	4	0	0	0	0	0
2	mmu-miR-139-5p	163	13	2	0	1424	5	0	0	188	2	0	0
2	mmu-miR-141	513	32	4	0	1394	23	0	0	0	0	0	0
2	mmu-miR-200a	1403	82	4	1	1594	73	3	0	438	65	4	1
2	mmu-miR-200c	613	41	4	0	1671	31	1	0	0	0	0	0
2	mmu-miR-322	266	17	3	0	1249	7	1	0	0	0	0	0
2	mmu-miR-322*	45	8	2	0	957	1	0	0	0	0	0	0
2	mmu-miR-429	380	26	4	0	1521	20	0	0	709	29	2	0
2	mmu-miR-449a	1934	359	15	10	1333	68	16	0	0	0	0	0
2	mmu-miR-449c	206	15	2	0	1342	13	1	0	0	0	0	0
2	mmu-miR-503	19	4	1	0	1280	1	0	0	203	0	0	0
2	mmu-miR-503*	199	13	2	0	801	4	1	0	0	0	0	0
3	mmu-miR-28*	136	11	0	12	878	4	0	0	0	0	0	0
3	mmu-miR-467a*	211	17	4	0	854	5	0	0	0	0	0	0
4	mmu-miR-127	10	1	0	0	1053	0	0	0	15	0	0	0
4	mmu-miR-135b	13	0	0	0	1237	0	0	0	394	0	0	0
4	mmu-miR-136	27	5	2	0	1024	0	0	0	125	0	0	0
4	mmu-miR-210	36	7	2	0	1309	0	0	0	22	0	0	0
4	mmu-miR-296-5p	10	3	0	0	1324	0	0	0	0	0	0	0
4	mmu-miR-298	6	0	0	0	1095	0	0	0	0	0	0	0
4	mmu-miR-299*	10	3	0	0	804	0	0	0	0	0	0	0
4	mmu-miR-301a	11	3	0	0	1479	0	0	0	0	0	0	0
4	mmu-miR-301b	13	3	1	0	1456	0	0	0	0	0	0	0
4	mmu-miR-335-3p	26	5	2	0	925	1	0	0	0	0	0	0
4	mmu-miR-337-5p	10	3	0	0	1274	0	0	0	0	0	0	0
4	mmu-miR-351	20	7	2	0	1235	0	0	0	385	2	0	0
4	mmu-miR-376a	10	3	0	0	1295	0	0	0	0	0	0	0
4	mmu-miR-376b*	15	3	0	0	797	0	0	0	0	0	0	0
4	mmu-miR-376c	11	2	0	0	1099	0	0	0	181	0	0	0
4	mmu-miR-379	10	1	0	0	1206	1	0	0	36	0	0	0
4	mmu-miR-382	8	0	0	0	1113	2	0	0	88	0	0	0
4	mmu-miR-409-3p	25	2	0	0	1198	1	0	0	0	0	0	0

4	mmu-miR-410	6	0	0	0	1148	0	0	0	342	0	0	0
4	mmu-miR-411	15	1	0	0	1279	0	0	0	66	0	0	0
4	mmu-miR-431	6	1	0	0	1125	0	0	0	77	0	0	0
4	mmu-miR-434-3p	12	3	0	0	1353	0	0	0	0	0	0	0
4	mmu-miR-434-5p	15	3	0	0	1280	0	0	0	0	0	0	0
4	mmu-miR-532-5p	16	4	2	0	1144	1	1	0	0	0	0	0
4	mmu-miR-539	6	1	0	0	901	0	0	0	354	0	0	0
4	mmu-miR-706	6	1	0	0	1559	0	0	0	0	0	0	0
5	mmu-miR-18a	3639	2700	1083	1038	1422	155	127	48	0	0	0	0
5	mmu-miR-106a	3585	2690	1257	1233	1537	139	100	39	0	0	0	0
5	mmu-miR-20b	3463	2550	1158	1042	1519	133	96	39	0	0	0	0
5	mmu-miR-20a	3630	2664	1012	1096	1528	141	98	37	0	0	0	0
5	mmu-miR-19a	2974	1842	565	505	1375	119	76	25	0	0	0	0
5	mmu-miR-17	3238	2140	710	643	1590	131	83	24	0	0	0	0
5	mmu-miR-19b	2906	1808	583	482	1369	108	67	23	716	221	132	41
5	mmu-miR-92a	2885	1564	348	321	1504	128	84	18	0	0	0	0
5	mmu-miR-466d-3p	3735	2326	231	270	1780	181	114	14	0	0	0	0
5	mmu-miR-15b*	2682	1311	202	204	914	71	32	1	0	0	0	0
5	mmu-miR-130b	5	1	0	0	1425	0	0	0	0	0	0	0
5	mmu-miR-134	3	0	0	0	1169	0	0	0	99	0	0	0
5	mmu-miR-138	10	3	2	0	1301	0	0	0	273	0	0	0
5	mmu-miR-149	5	2	0	0	1137	0	0	0	182	0	0	0
5	mmu-miR-182	7	2	0	0	1383	0	0	0	678	0	0	0
5	mmu-miR-214	4	1	0	0	1466	0	0	0	0	0	0	0
5	mmu-miR-214*	5	2	0	0	846	0	0	0	0	0	0	0
5	mmu-miR-323-3p	4	0	0	0	1403	0	0	0	0	0	0	0
5	mmu-miR-370	1	0	0	0	1279	0	0	0	166	0	0	0
5	mmu-miR-380-5p	2	0	0	0	1175	0	0	0	0	0	0	0
5	mmu-miR-433	7	3	0	0	1050	0	0	0	204	0	0	0
5	mmu-miR-483*	2	0	0	1	913	0	0	0	0	0	0	0
5	mmu-miR-485*	3	0	0	0	894	0	0	0	0	0	0	0
5	mmu-miR-494	6	3	0	0	1078	0	0	0	295	0	0	0
5	mmu-miR-495	5	0	0	0	1314	1	0	0	472	0	0	0
5	mmu-miR-672	13	5	0	0	1107	0	0	0	0	0	0	0
5	mmu-miR-690	13	4	1	0	1201	0	0	0	0	0	0	0
5	mmu-miR-696	132	6	0	2	1128	4	1	0	0	0	0	0
5	mmu-miR-708	5	2	0	0	1061	0	0	0	76	0	0	0
5	mmu-miR-709	4	2	0	0	1658	0	0	0	0	0	0	0
Total		184033	120400	69725	47532	148840	7463	5036	3066	10482	1315	703	330
