# Supplementary material for: MicroRNA Networks in Mouse Lung Organogenesis
Source: PLoS One. 2010 May 26;5(5):e10854. doi: 10.1371/journal.pone.0010854 (PMC2877109; doi:10.1371/journal.pone.0010854)
Supplement: Table S2 — Correlation analysis and direct target predication results of 117 significant miRNA by miRNA/protein correlations in mouse lung development. (0.12 MB PDF) [file pone.0010854.s002.pdf]

Table 2

A-Neg: Number of Negatively Correlated Targets (Coefficient cutoff<-0.7, -0.8 or -0.9))
B: Number of Computational Targets from miRBase
C: Number of direct miRNA targets through Overlapping of B and A-Neg

Cluster	miRNA	A Neg(0.7)	A Neg(0.8)	A Neg(0.9)	B	C Neg(0.7)	C Neg(0.8)	C Neg(0.9)
1	mmu-miR-126-3p	613	428	174	1234	28	12	0
1	mmu-miR-126-5p	684	442	331	1103	25	6	5
1	mmu-miR-146a	691	431	330	1423	20	10	7
1	mmu-miR-146b	755	506	332	1375	26	16	11
1	mmu-miR-150	723	473	335	1240	13	3	2
1	mmu-miR-155	686	448	323	1227	33	17	13
1	mmu-miR-191	610	424	318	1238	24	18	14
1	mmu-miR-195	644	415	322	1229	20	16	12
1	mmu-miR-222	704	461	330	1182	26	18	16
1	mmu-miR-223	636	428	324	1220	20	13	12
1	mmu-miR-24	612	351	69	1220	15	7	4
1	mmu-miR-24-2*	618	360	142	1165	8	5	2
1	mmu-miR-29a	661	373	56	1576	32	15	0
1	mmu-miR-29c	531	329	156	1557	25	18	10
1	mmu-miR-30a	844	605	115	1467	35	22	0
1	mmu-miR-30d	903	617	103	1432	37	20	0
1	mmu-miR-30e	861	372	162	1528	46	5	2
1	mmu-miR-31	904	593	28	1203	27	18	2
1	mmu-miR-34b-3p	647	380	151	1254	14	6	1
1	mmu-miR-489	707	450	323	1241	25	18	12
1	mmu-miR-133a	663	422	327	1356	18	10	7
1	mmu-miR-140	565	406	319	1281	21	16	15
1	mmu-miR-142-3p	605	420	321	1310	8	8	7
1	mmu-miR-145	654	423	314	1266	18	9	8
1	mmu-miR-16	633	460	60	1380	13	13	0
1	mmu-miR-181a	556	406	316	1374	19	8	8
1	mmu-miR-21	455	249	36	1148	19	14	5
1	mmu-miR-26a	739	469	30	1287	35	8	2
1	mmu-miR-26b	307	155	23	1254	21	9	0
1	mmu-miR-27a	642	418	30	1267	15	9	0
1	mmu-miR-30a*	540	327	97	1253	17	11	2
1	mmu-miR-30b	741	471	30	1517	32	20	0
1	mmu-miR-30c	724	510	109	1510	41	36	8
1	mmu-miR-30e*	553	362	100	1245	13	7	6
1	mmu-miR-328	583	414	318	1115	13	6	6
1	mmu-miR-34c*	536	329	135	983	24	14	2
1	mmu-miR-365	636	414	321	1385	12	6	5
1	mmu-miR-451	606	412	322	1278	25	14	14
1	mmu-miR-484	574	399	322	1199	16	14	14
1	mmu-miR-486	675	448	337	1436	19	10	9
1	mmu-miR-667	548	404	315	1283	12	12	11
1	mmu-let-7b	1043	409	127	1459	38	16	5
2	mmu-let-7c	137	69	34	1459	5	4	2
2	mmu-let-7d	121	67	13	1377	3	2	0
2	mmu-let-7e	126	68	1	1310	3	2	0
2	mmu-let-7g	137	74	24	1406	5	5	3

2	mmu-let-7i	86	36	3	1451	2	0	0
2	mmu-miR-139-5p	547	387	316	1424	16	12	8
2	mmu-miR-141	560	414	319	1394	8	7	5
2	mmu-miR-200a	581	404	319	1594	19	11	9
2	mmu-miR-200b	538	394	319	1601	18	17	12
2	mmu-miR-200c	539	390	314	1671	16	13	10
2	mmu-miR-322	519	370	312	1249	22	21	14
2	mmu-miR-322*	503	369	315	957	20	18	17
2	mmu-miR-429	561	407	314	1521	14	10	8
2	mmu-miR-449a	525	380	314	1333	16	13	13
2	mmu-miR-449c	511	386	314	1342	16	13	13
2	mmu-miR-503	472	378	300	1280	17	6	6
2	mmu-miR-503*	523	388	317	801	6	5	5
3	mmu-miR-28*	88	25	6	878	0	0	0
3	mmu-miR-467a*	709	454	333	854	8	2	2
4	mmu-miR-136	501	368	317	1024	8	6	5
4	mmu-miR-210	515	376	313	1309	18	15	11
4	mmu-miR-335-3p	497	365	314	925	5	4	0
4	mmu-miR-351	489	386	302	1235	11	10	6
4	mmu-miR-532-5p	496	374	311	1144	10	10	9
4	mmu-miR-127	428	357	11	1053	14	13	3
4	mmu-miR-135b	402	352	8	1237	12	12	3
4	mmu-miR-296-5p	445	374	300	1324	13	8	8
4	mmu-miR-298	453	358	25	1095	11	9	1
4	mmu-miR-299*	449	368	302	804	10	7	7
4	mmu-miR-301a	452	372	298	1479	27	23	17
4	mmu-miR-301b	463	376	306	1456	34	20	19
4	mmu-miR-337-5p	459	366	302	1274	12	8	8
4	mmu-miR-376a	441	361	303	1295	15	12	4
4	mmu-miR-376b*	448	365	301	797	1	1	1
4	mmu-miR-376c	442	352	14	1099	13	11	0
4	mmu-miR-379	413	354	11	1206	12	10	0
4	mmu-miR-382	437	60	5	1113	9	0	0
4	mmu-miR-409-3p	143	37	10	1198	3	2	2
4	mmu-miR-410	427	356	6	1148	14	12	0
4	mmu-miR-411	409	348	11	1279	9	5	1
4	mmu-miR-431	404	55	16	1125	9	2	2
4	mmu-miR-434-3p	462	347	302	1353	12	11	11
4	mmu-miR-434-5p	470	368	302	1280	14	6	2
4	mmu-miR-539	434	358	299	901	4	4	4
4	mmu-miR-706	469	356	11	1559	15	9	0
5	mmu-miR-106a	604	429	158	1537	37	25	19
5	mmu-miR-130b	471	369	308	1425	32	22	21
5	mmu-miR-134	415	349	4	1169	11	11	0
5	mmu-miR-138	524	387	314	1301	20	14	12
5	mmu-miR-149	504	369	304	1137	18	11	9
5	mmu-miR-15b*	422	243	80	914	6	4	2
5	mmu-miR-17	581	370	122	1590	44	30	19
5	mmu-miR-182	493	386	302	1383	17	6	6
5	mmu-miR-18a	509	304	115	1422	35	24	7
5	mmu-miR-19a	594	443	152	1375	33	22	12
5	mmu-miR-19b	580	370	122	1369	28	20	13
5	mmu-miR-20a	578	292	153	1528	38	19	18
5	mmu-miR-20b	1109	400	144	1519	65	31	16

5	mmu-miR-214	438	374	298	1466	21	14	10
5	mmu-miR-214*	460	381	302	846	4	4	3
5	mmu-miR-323-3p	420	355	11	1403	15	14	0
5	mmu-miR-370	442	355	4	1279	18	14	3
5	mmu-miR-380-5p	459	368	301	1175	19	16	7
5	mmu-miR-433	464	352	304	1050	4	3	3
5	mmu-miR-466d-3p	332	200	53	1780	20	9	9
5	mmu-miR-483*	541	68	18	913	9	1	1
5	mmu-miR-485*	417	345	16	894	7	7	0
5	mmu-miR-494	474	378	307	1078	12	9	9
5	mmu-miR-495	429	357	2	1314	11	11	0
5	mmu-miR-672	458	365	299	1107	14	14	3
5	mmu-miR-690	476	372	309	1201	15	13	13
5	mmu-miR-696	223	143	49	1128	16	9	5
5	mmu-miR-708	439	353	301	1061	11	9	9
5	mmu-miR-709	465	368	306	1658	19	13	13
5	mmu-miR-92a	579	347	119	1504	28	22	3
Total		61743	41774	22897	148840	2104	1345	755
