# Supplementary material for: MicroRNA Networks in Mouse Lung Organogenesis
Source: PLoS One. 2010 May 26;5(5):e10854. doi: 10.1371/journal.pone.0010854 (PMC2877109; doi:10.1371/journal.pone.0010854)
Supplement: Table S3 — A list of 30 miRNAs having direct targets identified by miRNA/mRNA correlation, with their respective corresponding top 10 GO biological process terms, all pathways and pathway-related target gene names. (0.04 MB PDF) [file pone.0010854.s003.pdf]

Table 3

Cluster	miRNA_ID	GO_Term	Pathway
1	miR-30a	transcription<br>regulation of transcription, DNA-dependent<br>cell cycle<br>mRNA processing<br>DNA repair<br>mitosis<br>RNA splicing<br>apoptosis<br>protein amino acid phosphorylation<br>response to DNA damage stimulus	mRNA_processing_binding_Reactome (Papola, Dhx9, Snrpb2, Hnrnpm, Eftud2, 2610027L16Rik)<br>RNA_transcription_Reactome (Polr3k, Gtf2a2, Gtf2h2)<br>Cell_Cycle_KEGG (Mad2l1, Cdc7, Hdac3, E2f6)<br>DNA_replication_Reactome (Cdc7)<br>G1_to_S_cell_cycle_Reactome (E2f6)<br>TGF_Beta_Signaling_Pathway (Tgfbr1)<br>Nuclear_Receptors (Cdc7)<br>GPCRDB_Other (Smo)
1	miR-30d	transcription<br>regulation of transcription, DNA-dependent<br>cell cycle<br>DNA repair<br>mRNA processing<br>mitosis<br>apoptosis<br>RNA splicing<br>double-strand break repair via homologous recombination<br>response to DNA damage stimulus	mRNA_processing_binding_Reactome (Papola, Dhx9, Snrpb2, Hnrnpm, 2610027L16Rik, Rbm8a, Tia1)<br>RNA_transcription_Reactome(Gtf2a2, Gtf2h2, Gtf2h4, Gtf2e2)<br>Cell_Cycle_KEGG (Dbf4, Mad2l1, Hdac3, E2f6)<br>DNA_replication_Reactome (Prim2, Dbf4, Rfc3)<br>G1_to_S_cell_cycle_Reactome (Prim2, E2f6)<br>TGF_Beta_Signaling_Pathway (Tgfbr1)<br>GPCRDB_Other (Smo)
1	miR-30e	transcription<br>regulation of transcription, DNA-dependent<br>cell cycle<br>mitosis<br>DNA repair<br>mRNA processing<br>apoptosis<br>protein amino acid phosphorylation<br>RNA splicing<br>protein folding	mRNA_processing_binding_Reactome (Papola, Dhx9, Snrpb2, Scyc1, Zfml, Hnrnpm, Snrpn, 2610027L16Rik, Rbm25, Tia1)<br>RNA_transcription_Reactome (Gtf2a2, Gtf2h2, Gtf2h4, Polr3k)<br>Cell_Cycle_KEGG (Dbf4, Mad2l1, Hdac3, E2f6)<br>DNA_replication_Reactome (Prim2, Dbf4)<br>G1_to_S_cell_cycle_Reactome (Prim2, E2f6)<br>TGF_Beta_Signaling_Pathway (Tgfbr1)<br>Ribosomal_Proteins (Rpl13a)<br>Proteasome_Degradation (Psmd7)
1	miR-24	transcription<br>regulation of transcription, DNA-dependent<br>DNA repair<br>protein folding<br>translation<br>proteolysis<br>protein polyubiquitination<br>cell cycle<br>DNA replication<br>cell division	mRNA_processing_binding_Reactome(Snrpc, Mrpl11, Elavl1, Ppie)<br>RNA_transcription_Reactome(Polr2c, Gtf2h2)<br>Cell_Cycle_KEGG(Plk1, E2f1)<br>DNA_replication_Reactome(Pola2, Rfc5)<br>G1_to_S_cell_cycle_Reactome(Pola2, E2f1)<br>Proteasome_Degradation(H2afx)
1	miR-24-2*	transport<br>transcription<br>regulation of transcription, DNA-dependent<br>DNA replication<br>RNA splicing<br>phosphatidylserine biosynthetic process<br>protein transport<br>protein amino acid phosphorylation<br>neuron migration<br>translation	mRNA_processing_binding_Reactome(Ddx20, Refbp2, Ncbp2, Sfrs11, Rbm16)<br>Apoptosis(Hells)<br>Cell_Cycle_KEGG(Plk1)<br>Krebs-TCA_Cycle(Idh2)

1	miR-29a	transcription<br>regulation of transcription, DNA-dependent<br>transport<br>protein amino acid phosphorylation<br>DNA repair<br>DNA replication<br>cell cycle<br>protein transport<br>mRNA processing<br>response to DNA damage stimulus	Cell_Cycle_KEGG(Ccna2, Espl1, Plk1, Hdac1, Pkmyt1, E2f1, Cdc7, Cdk2, Dbf4)<br>mRNA_processing_binding_Reactome(Ddx1, Snrpc, Eif4a3, Brca1, Cugbp1)<br>RNA_transcription_Reactome(Taf9, Gtf2e2)<br>DNA_replication_Reactome(Cdc7, Ddk2, Dbf4)<br>G1_to_S_cell_cycle_Reactome(Pkmyt1, E2f1, Cdk2)<br>Proteasome_Degradation(Psma1, Psmb7, H2afx)<br>Krebs-TCA_Cycle(Fh1, Idh2)<br>Nuclear_Receptors(Cdc7)<br>Electron_Transport_Chain(Slc25a5)<br>Apoptosis(Eif3k)<br>Ovarian_Infertility_Genes(Mlh1)
1	miR-29c	protein amino acid phosphorylation<br>regulation of transcription, DNA-dependent<br>transcription<br>proteolysis<br>translation<br>positive regulation of I-kappaB kinase/NF-kappaB -cascade<br>response to stress<br>response to drug<br>response to toxin<br>spermatogenesis	Cell_Cycle_KEGG(Cdk2, Ccna2)<br>mRNA_processing_binding_Reactome(Eif4e2)<br>RNA_transcription_Reactome(Gtf2e2)<br>DNA_replication_Reactome(Cdk2)<br>G1_to_S_cell_cycle_Reactome(Cdk2)<br>Striated_muscle_contraction(Tpm1)
1	miR-34b-3p	transcription<br>mRNA processing<br>regulation of transcription, DNA-dependent<br>RNA splicing<br>DNA replication<br>DNA repair<br>translation<br>apoptosis<br>protein amino acid phosphorylation<br>response to DNA damage stimulus	mRNA_processing_binding_Reactome(Rad21, Fbl, Tia1, Snrpa1, Syncrip, Prpf4b, Dhx9, Imp4, Sltm, Hnrnpa1, Taf15)<br>DNA_replication_Reactome(Pola1, Prim2, Pold3, Pcna, Dbf4)<br>Cell_Cycle_KEGG(E2f5, Pcna, Dbf4, Trp53)<br>G1_to_S_cell_cycle_Reactome(Pcna, Trp53, Prim2, E2f5)<br>RNA_transcription_Reactome(Tal9)<br>Translation_Factors(Eif2s3x)<br>Proteasome_Degradation(Psma1, Psmd12)<br>Apoptosis(Trp53)<br>Apoptosis_GenMAPP(Trp53)<br>Electron_Transport_Chain(Ndufa10)<br>G_Protein_Signaling(Prkar2b)<br>Calcium_regulation_in_cardiac_cells(Prkar2b)<br>Smooth_muscle_contraction(Prkar2b)<br>Nucleotide_Metabolism(Pola1)<br>Integrin-mediated_cell_adhesion_KEGG(Map2k6)<br>MAPK_Cascade(Map2k6)<br>Glycolysis_and_Gluconeogenesis(Ldha)
1	miR-31	transcription<br>regulation of transcription, DNA-dependent<br>DNA repair<br>mRNA processing<br>DNA replication<br>transport<br>RNA splicing<br>response to DNA damage stimulus<br>cell cycle<br>translation	Cell_Cycle_KEGG(Pkmyt1, Mcm7, Espl1)<br>G1_to_S_cell_cycle_Reactome(Pkmyt1, Mcm7)<br>DNA_replication_Reactome(Mcm7)<br>RNA_transcription_Reactome(Gtf2h3)<br>Electron_Transport_Chain(Atp5o)<br>Nucleotide_Metabolism(Srm)
1	miR-126-3p	transcription<br>regulation of transcription, DNA-dependent<br>mRNA processing<br>DNA repair<br>RNA splicing<br>cell cycle<br>translation<br>metabolic process<br>DNA damage checkpoint<br>DNA replication	mRNA_processing_binding_Reactome(Dhx15, Hnrnpa2b1, Ilf3, Xrn2, D19Bwg1357e, Igf2bp3, Brwd1)<br>DNA_replication_Reactome(Rfc2, Gmnn, Rfc1)<br>Cell_Cycle_KEGG(Atr)<br>Apoptosis(Casp3)<br>Apoptosis_GenMAPP(Casp3)<br>Krebs-TCA_Cycle(Pdk3, Idh2)<br>Apoptosis_KEGG(Casp3)<br>RNA_transcription_Reactome(Polr2e)<br>G_Protein_Signaling(Prkar2b)<br>Calcium_regulation_in_cardiac_cells(Prkar2b)<br>Smooth_muscle_contraction(Prkar2b)

2	miR-30a*	DNA replication<br>cell cycle<br>translation<br>mRNA processing<br>mitosis<br>RNA splicing<br>apoptosis<br>mitotic chromosome condensation<br>DNA repair<br>transport	mRNA_processing_binding_Reactome (Rbm17)<br>DNA_replication_Reactome (Mcm6, Orc4l)<br>G1_to_S_cell_cycle_Reactome (Mcm6, Orc4l)<br>Cell_Cycle_KEGG (Mcm6, Orc4l)<br>Translation_Factors (Eef1d, Etf1)<br>Proteasome_Degradation (Psmc6, Psmd1)<br>TGF_Beta_Signaling_Pathway(Tgfbr1)<br>Electron_Transport_Chain (Ndufa10)
2	miR-30b	transcription<br>regulation of transcription, DNA-dependent<br>cell cycle<br>mRNA processing<br>protein amino acid phosphorylation<br>mitosis<br>apoptosis<br>transport<br>DNA repair<br>RNA splicing	mRNA_processing_binding_Reactome (Snrpc, Snrpn, Rbm8a, Papola, Dhx9, Hnrnpm, Tia1)<br>RNA_transcription_Reactome (Polr3k, Gtf2h4, Gtf2e2, Gtf2h2)<br>Cell_Cycle_KEGG (Bub1b, Cdc7, Hdac3)<br>Wnt_Signaling (Fzd2, Racgap1)<br>DNA_replication_Reactome (Cdc7)<br>Nuclear_Receptors (Cdc7)<br>S1P_Signaling (Racgap1)<br>Proteasome_Degradation (Psmd12)<br>GPCRDB_Other (Fzd2)
2	miR-30c	transcription<br>regulation of transcription, DNA-dependent<br>cell cycle<br>mRNA processing<br>mitosis<br>DNA repair<br>apoptosis<br>transport<br>RNA splicing<br>protein amino acid phosphorylation	mRNA_processing_binding_Reactome(Snrpc, Scye1, Smc1a, Rbm8a, Papola, Rbm25, Dhx9, Hnrnpm, 2610027L16Rik)<br>RNA_transcription_Reactome (Gtf2h4, Polr3k, Gtf2h2)<br>Cell_Cycle_KEGG (Bub1b, Dbf4, Hdac3)<br>DNA_replication_Reactome (Dbf4)<br>Proteasome_Degradation (Psmd12)<br>GPCRDB_Other (Smo)<br>Cholesterol_Biosynthesis (Sqle)
2	miR-30e*	cell cycle<br>mitosis<br>mitotic chromosome condensation<br>DNA replication<br>mRNA processing<br>protein folding<br>translation<br>cell division<br>RNA splicing<br>apoptosis	mRNA_processing_binding_Reactome (Ssb, Scye1, Tsn, Rngtt, Sf3b2, Rbm17)<br>DNA_replication_Reactome(Mcm6, Orc6, Prim2)<br>G1_to_S_cell_cycle_Reactome(Mcm6, Orc6, Prim2)<br>Cell_Cycle_KEGG(Mcm6, Orc61)<br>Proteasome_Degradation(Psmc6, Psmd1)<br>Translation_Factors(Eif3i, Etf1)<br>TGF_Beta_Signaling_Pathway(Tgfbr1)<br>Electron_Transport_Chain(Ndufa10)<br>Pentose_Phosphate_Pathway(Rpe) Inflammatory Response Pathway (2610524H06Rik)
2	miR-26a	transcription<br>regulation of transcription, DNA-dependent<br>translation<br>cell cycle<br>protein amino acid phosphorylation<br>regulation of transcription<br>DNA damage checkpoint<br>mRNA processing<br>protein folding<br>cell division	mRNA_processing_binding_Reactome(Pabpc1, Rbm8a, G3bp1, Xrn2, Mki67ip, Rngtt, Cugbp1, Brwd1, Hnrpdl)<br>Cell_Cycle_KEGG(Ccne1, Chek1)<br>Apoptosis(Hells)<br>G1_to_S_cell_cycle_Reactome(Ccne1)<br>Translation_Factors(Eif5a, Pabpc1)<br>RNA_transcription_Reactome(Gtf2a2)
2	miR-26b	regulation of transcription, DNA-dependent<br>cell cycle<br>transcription<br>translation<br>regulation of transcription<br>mitosis<br>protein amino acid phosphorylation<br>transport<br>cell division<br>cell morphogenesis	mRNA_processing_binding_Reactome(Pabpc1, Rbm8a, G3bp1, Cugbp1)<br>Translation_Factors(Eif4a1, Eif5a, Pabpc1)<br>Cell_Cycle_KEGG(Ccne1, Mad2l1)<br>Apoptosis(Hells)<br>G1_to_S_cell_cycle_Reactome(Ccne1)<br>RNA_transcription_Reactome(Gtf2a2)

2	miR-34c*	transcription<br>regulation of transcription, DNA-dependent<br>DNA repair<br>translation<br>mRNA processing<br>translational initiation<br>DNA damage checkpoint<br>nuclear mRNA splicing, via spliceosome<br>apoptosis<br>response to DNA damage stimulus	mRNA_processing_binding_Reactome(Eif4e, Fbl, Tia1, Snrpa, Ilf3, Dhx9, Sltm, Hnrnpa1, Taf15)<br>Translation_Factors(Eif2s2, Eif2s3x, Eef1b2, Eif4e)<br>Cell_Cycle_KEGG(Dbf4, Atr)<br>RNA_transcription_Reactome(Tal9)<br>Proteasome_Degradation(Psma1, Psmd12)<br>DNA_replication_Reactome(Dbf4)<br>Electron_Transport_Chain(Ndufa10)
2	miR-16	transport<br>mRNA processing<br>modification-dependent protein catabolic process<br>protein amino acid phosphorylation<br>apoptosis<br>rRNA processing<br>RNA splicing<br>cell cycle<br>protein transport<br>negative regulation of transcription	mRNA_processing_binding_Reactome(Ddx1, Fbl, Thoc4, Sfrs9, Rbm9, Tarbp2, Hnrnpl, Pcbp4, Xrn2, Ppp1r14b)<br>Cell_Cycle_KEGG(Ccne1)<br>G1_to_S_cell_cycle_Reactome(Ccne1)<br>RNA_transcription_Reactome(Polr2h)<br>Proteasome_Degradation(Psmd13)<br>Pentose_Phosphate_Pathway(G6pd2)<br>Nuclear_Receptors(Rarb)
2	miR-21	mRNA processing<br>transcription<br>regulation of transcription, DNA-dependent<br>RNA splicing<br>cell cycle<br>protein amino acid phosphorylation<br>DNA repair<br>multicellular organismal development<br>mitotic chromosome condensation<br>DNA replication	mRNA_processing_binding_Reactome(Npm1, Snrpa1, Sfrs9, Pspc1, Rbmx2, Rbm25, Rnps1, Rbm17)<br>G1_to_S_cell_cycle_Reactome(Rpa2, Ccne1, Pcna)<br>Cell_Cycle_KEGG(Ccne1, Pcna2)<br>DNA_replication_Reactome(Rpa2, Pcna)<br>G_Protein_Signaling(Gng2)<br>Calcium_regulation_in_cardiac_cells(Gng2)<br>Smooth_muscle_contraction(Gng2)<br>RNA_transcription_Reactome(Polr2e, Gtf2e2)<br>Proteasome_Degradation(Psmd7)<br>Apoptosis(Birc5)
2	miR-27a	protein polyubiquitination<br>mRNA processing<br>DNA repair<br>protein folding<br>inner cell mass cell proliferation<br>nuclear mRNA splicing, via spliceosome<br>mRNA export from nucleus<br>RN<br>translational elongation<br>transcription<br>multicellular organismal development	mRNA_processing_binding_Reactome(Rbpms2, Nxf1)<br>RNA_transcription_Reactome(Polr1a)<br>Nucleotide_Metabolism(Srm)
3	mmu-let-7b	transcription<br>regulation of transcription, DNA-dependent<br>DNA repair<br>mRNA processing<br>regulation of translation<br>cell cycle<br>mitosis<br>cell division<br>RNA polyadenylation<br>RNA 3'-end processing	mRNA_processing_binding_Reactome(Tarbp2, Papola, Dhx8)<br>Apoptosis(Map3k1, Myc)<br>Translation_Factors(Eif4g1)<br>Apoptosis_GenMAPP(Map3k1, Myc)<br>G1_to_S_cell_cycle_Reactome(Myc)<br>Cholesterol_Biosynthesis(Hmgcs1)<br>Wnt_Signaling(Myc)<br>MAPK_Cascade(Map3k1)

7	miR-17	apoptosis<br>immune response<br>protein modification process<br>protein ubiquitination<br>fatty acid metabolic process<br>positive regulation of transcription<br>ganglioside biosynthetic process<br>transcription<br>regulation of transcription, DNA-dependent<br>protein amino acid dephosphorylation	Proteasome_Degradation (Ubc)<br>TGF_Beta_Signaling_Pathway (Smad6)<br>Prostaglandin_synthesis_regulation (Tbxas1)<br>Eicosanoid_Synthesis (Tbxas1)
7	miR-18a	immune response<br>regulation of transcription, DNA-dependent<br>lipid biosynthetic process<br>fatty acid biosynthetic process<br>protein amino acid phosphorylation<br>transcription<br>intracellular signaling cascade<br>apoptosis<br>G-protein coupled receptor protein signaling-pathway<br>multicellular organismal development	Calcium_regulation_in_cardiac_cells (Adrb1, Prkce)<br>Wnt_Signaling (Prkce)<br>G_Protein_Signaling (Prkce)<br>Smooth_muscle_contraction (Prkce)<br>TGF_Beta_Signaling_Pathway (Smad6)<br>Eicosanoid_Synthesis (Alox5)<br>GPCRDB_Other (Adrb1)<br>Monoamine_GPCRs (Adrb1)<br>GPCRDB_Class_A_Rhodopsin-like (Adrb1)
7	miR-19a	protein amino acid dephosphorylation<br>dephosphorylation<br>protein amino acid phosphorylation<br>fatty acid metabolic process<br>lipid metabolic process<br>prostaglandin metabolic process<br>endocytosis<br>regulation of endocytosis<br>apoptosis<br>multicellular organismal development	Prostaglandin_synthesis_regulation (Hpgd)<br>mRNA_processing_binding_Reactome (Tmem106a)
7	miR-20a	regulation of transcription, DNA-dependent<br>transcription<br>fatty acid metabolic process<br>apoptosis<br>signal transduction<br>protein amino acid ADP-ribosylation<br>anti-apoptosis<br>cytokine-mediated signaling pathway<br>B cell homeostasis<br>B cell mediated immunity	mRNA_processing_binding_Reactome (Cpeb4)<br>TGF_Beta_Signaling_Pathway (Smad6)<br>GPCRDB_Other (S1pr1)<br>Nuclear_Receptors (Rora)<br>S1P_Signaling (S1pr1)<br>Small_ligand_GPCRs (S1pr1)<br>Circadian_Exercise (Cldn5)
7	miR-19b	protein amino acid phosphorylation<br>apoptosis<br>protein amino acid dephosphorylation<br>dephosphorylation<br>angiogenesis<br>intracellular signaling cascade<br>fatty acid metabolic process<br>signal transduction<br>lipid metabolic process<br>prostaglandin metabolic process	Calcium_regulation_in_cardiac_cells (Prkce)<br>Wnt_Signaling (Prkce)<br>G_Protein_Signaling (Prkce)<br>Smooth_muscle_contraction (Prkce)<br>mRNA_processing_binding_Reactome (Tmem106a)<br>Prostaglandin_synthesis_regulation (Hpgd)
7	miR-92a	signal transduction<br>multicellular organismal development<br>metabolic process<br>carbohydrate metabolic process<br>positive regulation of mesenchymal cell proliferation<br>negative regulation of transcription from-RNA polymerase II promoter<br>pre-B cell differentiati<br>intracellular signaling cascade<br>regulation of transcription, DNA-dependent<br>transcription	G_Protein_Signaling (Gna14)<br>TGF_Beta_Signaling_Pathway (Smad6)<br>Integrin-mediated_cell_adhesion_KEGG (Itgb3)

7	miR-106a	regulation of transcription, DNA-dependent<br>transcription<br>fatty acid metabolic process<br>protein amino acid phosphorylation<br>protein amino acid ADP-ribosylation<br>lipid metabolic process<br>metabolic<br>protein modification process<br>protein ubiquitination<br>positive regulation of transcription	Proteasome_Degradation (Ubc)<br>TGF_Beta_Signaling_Pathway (Smad6)<br>Prostaglandin_synthesis_regulation (Tbxas1)<br>Nuclear_Receptors (Rora)<br>Eicosanoid_Synthesis (Tbxas1)
7	miR-20b	regulation of transcription, DNA-dependent<br>transcription<br>fatty acid metabolic process<br>protein modification process<br>protein ubiquitination<br>positive regulation of transcription<br>anti-apoptosis<br>leukotriene metabolic process<br>response to organic substance<br>drug metabolic process	Proteasome_Degradation (Ubc)<br>Eicosanoid_Synthesis (Tbxas1, Alox5)<br>TGF_Beta_Signaling_Pathway (Smad6)<br>Prostaglandin_synthesis_regulation (Tbxas1)
7	miR-466d-3p	cell adhesion<br>ureteric bud development<br>microtubule-based movement<br>retrograde vesicle-mediated transport, Golgi - to ER<br>response to external stimulus<br>homophilic cell adhesion<br>regulation of transcription, DNA-dependent<br>transcription<br>metabolic process<br>multicellular organismal development	None
